# Supplementary material for: Development of Biocompatible Digital Light Processing Resins for Additive Manufacturing Using Visible Light-Induced RAFT Polymerization
Source: Polymers (Basel). 2024 Feb 8;16(4):472. doi: 10.3390/polym16040472 (PMC10893283; doi:10.3390/polym16040472)
Supplement: Supplementary file 1 [file polymers-16-00472-s001.zip › polymers-2838395-supplementary.pdf]

polymers

MDPI

Article

Development of biocompatible digital light processing resins for additive manufacturing using visible light-induced RAFT polymerization

Supplementary Material

Optimization of resin printability.

As is observed in **Figure S1-a**, when the model contains internal complex structures (simple and TPMS channels), a lack of printing resolution is observed in the obtained samples. Thus, a light absorber, Rose Bengal (RB), was added to the resin to overcome this issue and increase printing resolution and definition (printability). In **Figure 1a**, it is possible to observe the results obtained for the samples once the RB was added to the mixture, showing improved outcomes, thus forming micrometric channels with high resolution and almost no imperfections for two models designed with either a gyroid or diamond-like internal structure. **Figure S1-b** shows some results obtained when printing the different types of TPMSs (gyroid and diamond). The CAD models and some photographs and micrographs of the pieces are shown, demonstrating the accuracy of the printing process.

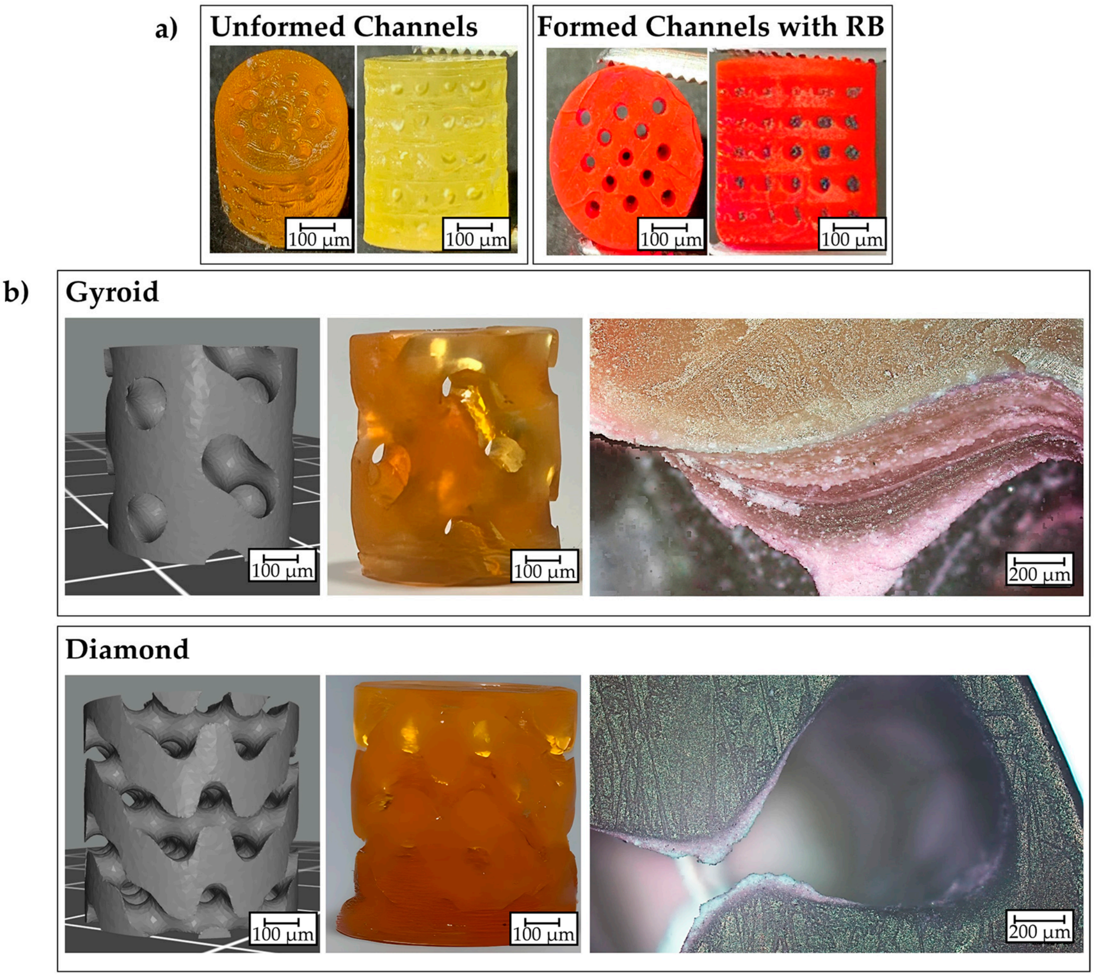

Figure S1. a) Photographs of printed structures with and without RB as photo-absorber. b) STL model (left), printed model (center), and optical microscopy (right) of the TPMS structures. Above: cylindrical model with gyroid internal structure. Below: cylindrical model with internal diamond structure (the dimensions of the CAD model: 10 mm x 10 mm).

Rheological characterization of the resin

A process that could be helpful to support resin printability is forming the so-called axisymmetric fluid bridge between two plates or liquid surfaces. This process is essential for applications in microfluidics and mass transport through porous media. In particular, the Bond (Bo) or Eötvös (Eo) number (eq. S1) is a dimensionless quantity that is used to determine if this bridge is stable or not. It is based on comparing the gravitational forces and the forces related to the surface tension of the fluid.

$$B_o = \frac{|\Delta\rho\, g|\, 2L^2}{\gamma_L} \quad \text{(S1)}$$

Where $\Delta\varrho$ is the difference in density between the liquid and the surrounding media, and in this case, the surrounding media is air with a negligible density, thus $\Delta\varrho \sim \varrho_{fluid}$. The Bond number also depends on g (gravity acceleration constant), L, the characteristic length, and γ_L, which is the surface tension of the fluid. A high value of B_o indicates that the system is relatively unaffected by surface tension effects. In contrast, a low value (typically less than one) suggests that the surface tension of the fluid dominates the system, thus forming stable and axisymmetric liquid bridges.
The values of the dynamical viscosity, surface tension, density, and Bond number are tabulated in **Table S1** for each resin analyzed. The results will also be compared to those obtained from the biocompatible commercial resin used as the gold standard (Zortrax Raydent Crown and Bridge). The characteristic length used in this case was the half-width of the printing layer thickness (25 µm).

Table S1. Rheological parameters for the three analyzed resins: gold standard, PDAD-8, and PHHC-3. The Bond number was determined using these values and **eq. S1**.

Resin	Dynamical viscosity (mPa·s)	Surface tension (mN/m)	Density (g/mL)	Bond number (B_o)
Gold standard	530.46 ± 0.0126	30.75 ± 1.70	1.01 ± 0.078	1.01 x 10^{-4}
PDAD-8	19.90 ± 0.0048	49.19 ± 0.40	1.12 ± 0.021	6.97 x 10^{-5}
PHHC-3	8.80 ± 0.0053	44.69 ± 2.63	1.11 ± 0.011	7.61 x 10^{-5}

The surface tension and density values for the resins PDAD-8 and PHHC-3 are remarkably similar to those reported for the gold standard resin (biocompatible commercial resin). In the case of dynamical viscosity, the values measured are larger for the gold standard resin. However, they are not a problem since they are still below the upper limit reported by Becker et al. On the other hand, the Bond numbers are close to zero (Bo ~ 0) for all the resins, which means that the liquid bridge formed between the liquid interface and the metallic plate surface is stable, considering the characteristic length as the half-separation between these surfaces (printing layer thickness = 25 µm). This result indicates that these resins are, a priori, excellent candidates to be printed via DLP technology. Using these values, it is possible to estimate that the maximum possible length of the liquid bridge to maintain a stable condition (Bo ~ 1) is approximately 3000 µm, which means that a separation over 3 mm between the liquid interface and the metallic plate surface will produce an unstable bridge, which will eventually collapse and separate from the plate, more than enough to be used as resin for DLP printing technology.
Another critical factor that significantly affects the printing process of DLP technology is the adhesion force between the resin and the printing plate. An indirect way to characterize this adhesion is via the contact angle between the resin and the plate. In this case, the contact angles of the PDAD-8 resin with the metallic plate are 9.73 ± 1.51° and 7.87 ± 1.95° for the PHHC-3 resin, while for the gold standard resin, the contact angle is slightly higher, 19.85 ± 1.17°. All these contact angles are low enough (< 30°), which means that the resins are suitable for use as a base material in DLP printing since they will establish excellent contact with the support and, as a result, upon UV irradiation, adhere firmly to the surface. This will prevent undesirable detachment during the printing process. **Figure S2** shows some contact angles measured over the metallic printing plate.

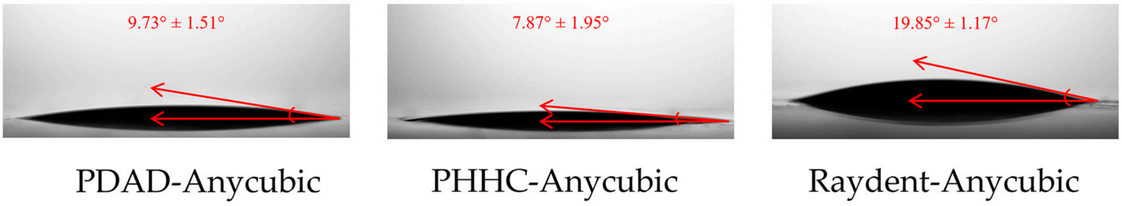

Figure S2. Contact angle images of the three analyzed resins (PDAD, PHHC, and gold standard) over the metallic printing plate.

Chemical and structural characterization of the bioceramic particles

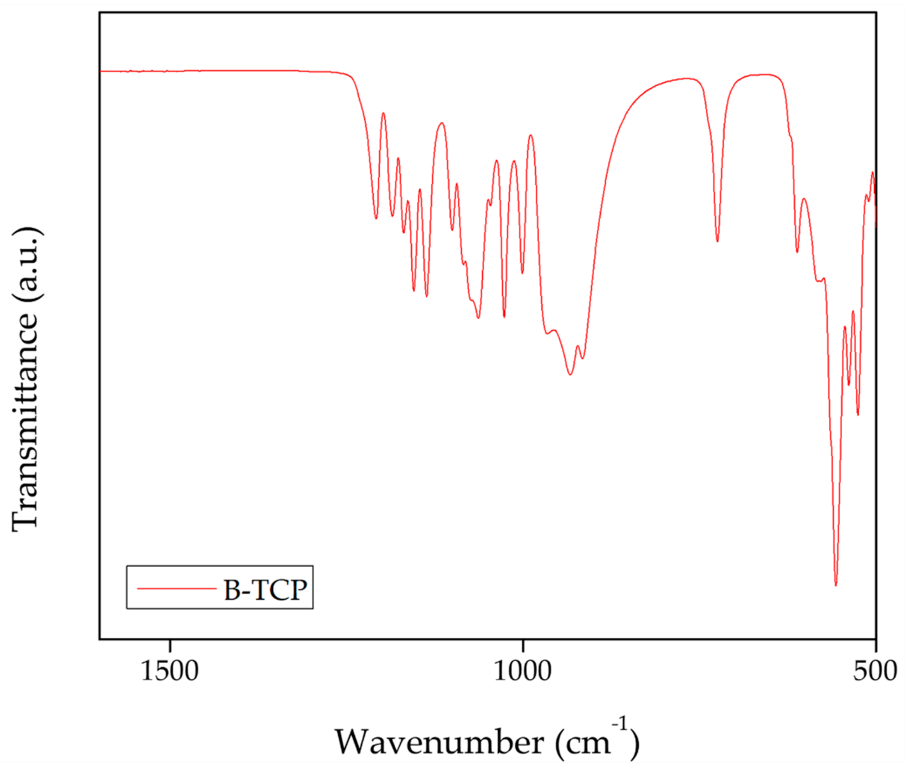

Figure S3. FT-IR spectra of the synthesized β-TCP powder.

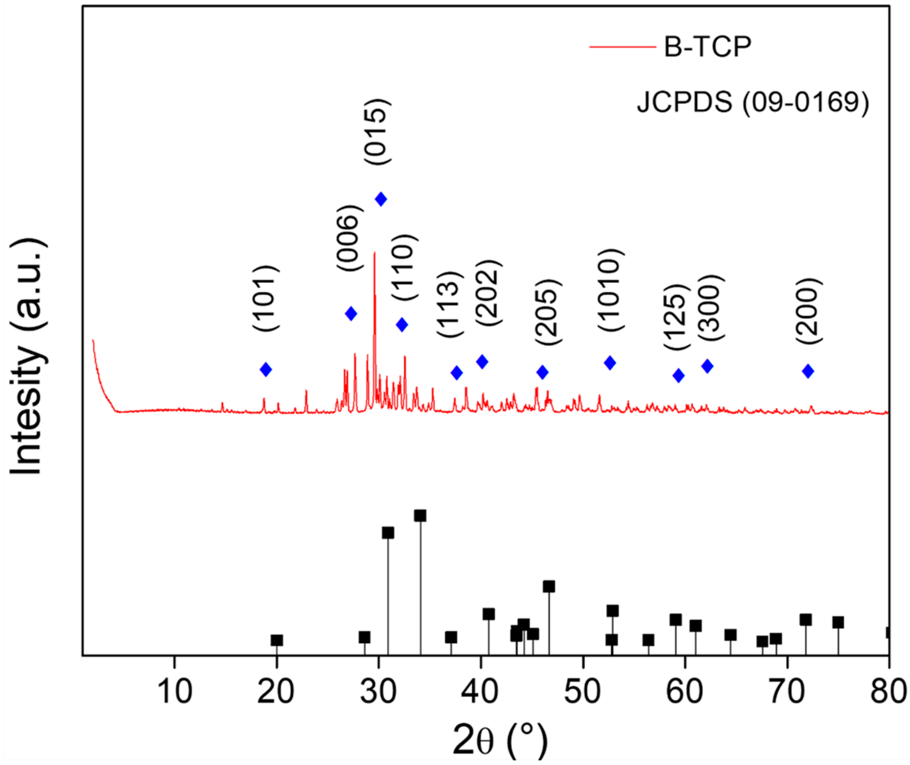

Figure S4. XRD spectra of the synthesized β-TCP powder.
